# Quality Assessment of Artemether-Lumefantrine Samples and Artemether Injections Sold in the Cape Coast Metropolis

**DOI:** 10.1155/2016/8602619

**Published:** 2016-02-23

**Authors:** James Prah, Elvis Ofori Ameyaw, Richmond Afoakwah, Patrick Fiawoyife, Ernest Oppong-Danquah, Johnson Nyarko Boampong

**Affiliations:** ^1^Department of Biomedical and Forensic Sciences, University of Cape Coast, Cape Coast, Ghana; ^2^University of Cape Coast Hospital, Cape Coast, Ghana; ^3^Ernest Chemist Manufacturing Limited, P.O. Box 3345, Accra-North, Ghana

## Abstract

Most prescribers and patients in Ghana now opt for the relatively expensive artemether/lumefantrine rather than artesunate-amodiaquine due to undesirable side effects in the treatment of uncomplicated malaria. The study sought to determine the existence of substandard and/or counterfeit artemether-lumefantrine tablets and suspension as well as artemether injection on the market in Cape Coast. Six brands of artemether-lumefantrine tablets, two brands of artemether-lumefantrine suspensions, and two brands of artemether injections were purchased from pharmacies in Cape Coast for the study. The mechanical properties of the tablets were evaluated. The samples were then analyzed for the content of active ingredients using High Performance Liquid Chromatography with a variable wavelength detector. None of the samples was found to be counterfeit. However, the artemether content of the samples was variable (93.22%−104.70% of stated content by manufacturer). The lumefantrine content of the artemether/lumefantrine samples was also variable (98.70%–111.87%). Seven of the artemether-lumefantrine brands passed whilst one failed the International Pharmacopoeia content requirements. All brands of artemether injections sampled met the International Pharmacopoeia content requirement. The presence of a substandard artemether-lumefantrine suspension in the market should alert regulatory bodies to be more vigilant and totally flush out counterfeit and substandard drugs from the Ghanaian market.

## 1. Introduction

The morbidity and mortality associated with malaria make it a major health problem in tropical and subtropical countries. According to the National Malaria Control Program (NMCP) annual report [1], malaria has been a major cause of poverty and low productivity accounting for about 32.5% of all outpatient attendances and 48.8% of under 5 years admissions in Ghana. It is therefore important that antimalarial medications administered are genuine and of high quality. The World Health Organization (WHO) first endorsed artemisinin-based combination therapy (ACT) for the treatment of malaria in 2004 and recommended a switch to ACTs as the first line malaria treatment in 2005 [2]. In compliance with WHO recommendation, Ghana replaced chloroquine with ACTs as the first line treatment for uncomplicated malaria in 2005. Since 2014, three ACTs have been selected for use nationally in Ghana to treat uncomplicated malaria. These are artesunate-amodiaquine (AA), artemether-lumefantrine (AL), and dihydroartemisinin-piperaquine (DHAP). In case of severe malaria, available options in order of preference are intravenous (IV)/intramuscular (IM) artesunate, IM artemether, and IV/IM quinine [3]. Even though the first line of treatment of uncomplicated malaria is artesunate/amodiaquine (AA) in Ghana, due to widely reported cases of unbearable side effects, most prescribers and patients are now opting for the relatively expensive artemether/lumefantrine (AL). In view of this, the Ghanaian market is dominated by this product (AL) with many generics available [4].

In recent years, the sources of supply, distribution, and quality of antimalarial drugs in various countries have recently engaged the serious attention of researchers. This is because poor quality antimalarials, either as counterfeit or as substandard artemisinins, have been reported in different parts of the world. The WHO has estimated that 25% of all the medicines in developing countries are counterfeit and this figure is projected to be as high as 40–50% in Nigeria and Pakistan [5]. Counterfeit or substandard artemisinin-based antimalarials and quinine may cause serious injury or death to patients because of mistreatment [6]. These may also cause a reduction in public confidence in medicines which could result in reduced intake of potentially life-saving medicines [7]. Substandard and counterfeit drugs have been implicated in the development of drug resistant strains of the malaria parasite [8]. Some studies in Ghana [9] have proved the existence of substandard artemisinin-based drugs in the country.

This study was therefore aimed at assessing the quality of the various brands of artemether-lumefantrine tablets and suspensions and two artemether injections available at pharmacies in Cape Coast, in the Central Region of Ghana.

## 2. Materials and Methods

### 2.1. Drugs and Reagents

Pure artemether and lumefantrine powders were from Sigma-Aldrich Inc. (St. Louis, MO, USA). Artemether-lumefantrine tablets coded AL 1 and AL 3 were manufactured in Ghana and AL 2, AL 6, and AL 7 in India, whilst AL 4 and AL 5 tablets were manufactured in China. Artemether-lumefantrine suspensions coded AL 7 and AL 8 were manufactured in India and Ghana, respectively. Artemether injection coded AT 1 was manufactured in India whilst AT 2 was manufactured in Ghana.

### 2.2. Drugs Sampling

A list of all licensed private pharmacy and chemical shops located in Cape Coast was made. Shops were selected at random using systematic sampling technique with a sampling interval of two. At each selected shop, a study agent who posed as a customer purchased any available artemether-lumefantrine tablet and suspension as well as any available brand of artemether injection. In all five blister packs each of the six brands of artemether-lumefantrine tablets, two brands of artemether-lumefantrine suspensions, and two brands of artemether injections manufactured by various pharmaceutical companies in Ghana (4) and abroad (6) were purchased between January and March, 2014. All the brands sampled had a remaining shelf life of at least one year at the time of sampling.

Although there are many experimental methods described in the literature for the assay of artemether-lumefantrine samples and artemether injection, High Performance Liquid Chromatography (HPLC) with a variable wavelength detector (HPLC Agilent Technologies 1200 Series, Germany) which has been widely used in such analysis due to its sensitivity, accuracy, and precision was employed in this study.

### 2.3. Visual Inspection

Prior to the more rigorous quantitative tests, visual inspection of packaging and dosage form was employed as a quick means of checking the quality or otherwise of the samples. The packaging was checked for correct and legible labelling of active ingredients and strength, expiration date, batch number, manufacturer, and country of origin. The study did not go as far as forensic examination of trademarks, product designs, or holograms. The appearance of the samples was also examined for discolouration, chippings, or excessive powder.

### 2.4. Uniformity of Weight and Disintegration of Tablets

Twenty (20) tablets taken at random from each sample were weighed individually and then weighed together. The average weight of each sample was determined. The deviation of each of the individual tablets in each sample from the average weight of the sample was also determined.

Disintegration times of six randomly selected tablets were determined in distilled water at 37 ± 0.5°C using Pharmatest disintegration test apparatus (Type PTZ-AUTO2, Germany).

### 2.5. Breaking Strength and Friability of Tablets

The breaking strength of ten randomly selected tablets was determined using Pharmatest hardness tester (Type PTB111E-500, Germany).

The friability (%) of twenty randomly selected tablets was determined using Copley friabilator (Type FRV 2000) operated at 25 rpm for 4 minutes.

### 2.6. Preparation of Standard Artemether

Standard artemether solution was prepared by dissolving 50 mg of pure artemether powder in 50 mL of acetonitrile and sonicated for 15 minutes at a frequency of 33 KHz. The resulting solution was made up to 100 mL with acetonitrile. The solution was then filtered through a syringe membrane filter and 25 *μ*L per injection was used for analysis.

### 2.7. Preparation of Standard Lumefantrine

Hundred milligrams of pure lumefantrine powder was dissolved in 50 mL of 0.1 M methanolic HCl and sonicated for 15 minutes at a frequency of 33 KHz. The resulting solution was made up to 100 mL with 0.1 M methanolic HCl. The solution was then filtered through a syringe membrane filter and 25 *μ*L per injection was used for analysis.

### 2.8. Preparation of Samples for Assay of Artemether

Twenty tablets of each artemether-lumefantrine samples were weighed and the average weight was determined. The tablets were crushed with a pestle in a mortar into powder. For the artemether-lumefantrine suspensions, the samples were reconstituted according to manufacturer's instruction and sonicated for 30 minutes. An amount of the powder or suspension equivalent to 50 mg of artemether was weighed into a 100 mL volumetric flask. Fifty millilitres of acetonitrile was added to the powder or suspension and the mixture was sonicated for 15 minutes at a frequency of 33 KHz. The resulting solution was made to 100 mL with acetonitrile. The solution was then mixed and filtered through a Whatman filter paper and the filtrate passed through a syringe membrane filter. A volume of 25 *μ*L per injection was used for analysis. The whole procedure was repeated for all the artemether-lumefantrine tablet and suspension samples.

### 2.9. Preparation of Samples for Assay of Lumefantrine

An amount of artemether-lumefantrine powder or suspension equivalent to 100 mg of lumefantrine was weighed into a 100 mL volumetric flask and was dissolved in 50 mL of 0.1 M methanolic HCl. The mixture was sonicated for 15 minutes at a frequency of 33 KHz. The resulting solution was made up to 100 mL with 0.1 methanolic HCl. The solution was then mixed and filtered through a Whatman filter paper and the filtrate passed through a syringe membrane filter. A volume of 25 *μ*L per injection was used for analysis. The whole procedure was repeated for all the artemether-lumefantrine tablet and suspension samples.

### 2.10. Preparation of the Artemether Injection Sample

The concentration of the artemether injection sample was 80 mg/mL. Therefore 0.625 mL equivalent to 50 mg of artemether was measured and added to 50 mL of acetonitrile in a 100 mL volumetric flask. The mixture was sonicated for 15 minutes at a frequency of 33 KHz. The resulting solution was made up to 100 mL with acetonitrile and then mixed and filtered through a syringe membrane filter. A volume of 25 *μ*L per injection was used for analysis.

### 2.11. Chromatographic Conditions

The actual amounts of artemether and lumefantrine in the samples were determined by comparing the areas of the samples (repeated twice for each sample) and standard peaks (repeated twice) as recorded on the chromatograms obtained by High Performance Liquid Chromatography (HPLC) using the following chromatographic conditions for all samples: Zorbax SB–C18 (4.6 mm × 250 mm, 5 *μ*m), mobile phase of 70% acetonitrile and 30% distilled water, injection volume of 25 *μ*L, and a flow rate of 1.5 mL/min. A wavelength of 216 nm was used to detect artemether and 335 nm for lumefantrine. A temperature of 28°C was employed.

The retention times for artemether and lumefantrine were 3.59 min and 0.41 min, respectively. The run time for both compounds was 6 min.

### 2.12. Data Analysis and Reporting

GraphPad Prism for Windows version 5 (GraphPad Software, San Diego, CA, USA) was used for all statistical analyses. Data are expressed as mean ± SEM and percentages. Mean values were subjected to ANOVA and *P* < 0.05 was considered statistically significant.

We used the MEDQUARG guidelines for reporting field surveys of the quality of medicines as proposed by Newton et al. (2009) [10] with minor modifications.

## 3. Results

### 3.1. Uniformity of Weight and Disintegration of Tablets

Visual inspection did not reveal any false labelling. All the samples were registered with the Food and Drug Authority. The shelf life of the samples ranged from 2 to 4 years. All tablets were found to be uncoated.  Table 1 shows the country of manufacture of all samples and some mechanical properties of the AL tablets. The percentage weight deviation of the various brands of AL tablets from their respective mean weights was less than 10% (Table 2). All the tablets disintegrated in aqueous medium in less than 15 minutes (900 seconds) (range: 554 ± 2.2–866 ± 5.2). However, the mean disintegration time for AL 6 was significantly (*P* < 0.001)* longer* than all the disintegration time of AL 1–5.

### 3.2. Breaking Strength and Friability of Tablets

The breaking strength of the tablets ranged from 3.4 to 5.3 kPa (Table 1). The percentage friability for all the AL tablets tested was lower than 1% w/w (range: 0.01–0.23% w/w).

### 3.3. Active Content Test for Artemether and Lumefantrine

Quantitative tests on all the different brands of AL and artemether injections proved the existence of artemether and lumefantrine in the AL samples and artemether in the artemether injections.  Table 3 depicts the actual content of artemether and lumefantrine in the samples and artemether in the artemether injection. The percentage artemether in the samples ranged from 98.04 to 102.82 in the AL tablet and suspension samples (Table 4) and for the artemether injections (AT) (Table 4). The artemether content was 99.92% in AT 1 and 98.17% in AT 2.

The percentage of lumefantrine ranged from 98.70 to 111.87. AL 8 suspensions recorded the highest artemether content, that is, 102.82%, whilst AL 5 tablet had the lowest but acceptable artemether content of 98.04%. AL 4 tablet recorded the lowest but acceptable lumefantrine content of 98.70% whilst the AL 8 suspension studied had the highest lumefantrine content of 111.87% (Table 4) which is above the maximum acceptable concentration of 110% (International Pharmacopoeia, 2003).

## 4. Discussion

Physical examination of the product packaging and the samples (tablets, suspensions, and injection) did not give any indication of fakery or counterfeiting. Counterfeit drugs have been defined by the WHO as drugs which are deliberately and fraudulently mislabelled with respect to identity or source and occur with branded and generic products and may include products with the correct ingredients but fake packaging, with the wrong ingredients, without active ingredients, or with insufficient active ingredients [11]. On the other hand, substandard drugs are genuine products that do not meet the specified quality standards [11]. All the tablets possessed appropriate mechanical properties in terms of weight uniformity, breaking strength, friability, and disintegration time (Table 1). The uniformity of weight test is one way of determining whether proper mixing or blending of ingredients occurred during manufacture. All the samples passed the test, although one tablet of AL 3 deviated by more than 5%. This means that all the samples conformed to the British Pharmacopoeia [12] specification of not more than 5% deviation for more than two of the individual masses and no deviation by more than 10% of the average mass of the tablets.

For a drug to be absorbed from a solid dosage form after oral administration, it must first be in solution, and the first important step toward this condition is a process known as disintegration. From Table 1, it can be seen that all the tablets tested passed the disintegration test as specified by the British Pharmacopoeia [12] which states that, for uncoated tablets, disintegration should occur within 15 minutes. All the samples also passed the percentage friability test of not more than 1% as specified by the British Pharmacopoeia [12]. It could thus be inferred that all the AL tablets studied could withstand abrasion without loss of tablet integrity and could disintegrate readily in an aqueous medium such as the gastrointestinal tract.

Quantitative analysis using HPLC-VWD showed 7 (87.50%) of the AL samples passing and 1 (12.50%) failing the International Pharmacopoeia [11] requirement which specifies that artemether-lumefantrine samples should contain not less than 90.00% and not more than 110.00% of the amount of artemether and lumefantrine stated on the label. The one AL sample that did not meet the International Pharmacopoeia [11] requirement had a higher than accepted lumefantrine content. This is unacceptable because the role of lumefantrine in a combination therapy is very important and cannot be compromised. Although the lumefantrine enjoys remarkable safety profile, it however poses side effects such as prolongation of the electrocardiographic QT interval; palpitation and dizziness occur [13] and therefore overdosage must be avoided. On the whole, AL samples manufactured abroad were of similar quality as those produced in Ghana.

The artemether content of the injection sample AT 1 was 99.92% and that of AT 2 was 98.17% of indicated amount on label. This means the samples met the International Pharmacopoeia [11] requirement which states that artemether injection should contain not less than 95.0% and not more than 105.0% of the artemether stated on the label. Since artemether injection is used in the treatment of complicated malaria, having the right amount of artemether content as found in this study is good for the management of malaria in Ghana. Even though no counterfeit drug was identified in the study, the observance that one AL sample was substandard means that drug regulatory bodies in Ghana and other African countries need to be vigilant and undertake routine assessment of the quality of AL and other artemisinin products on the market.

## 5. Conclusion

Out of 8 artemether-lumefantrine brands and two artemether injections sampled, none was found to be fake or counterfeit. One of the AL suspensions was found to be substandard. Though most of the samples were of good standard, the regulatory bodies must continue to be vigilant in order to flush out totally counterfeit and substandard drugs.

## Figures and Tables

**Table 1:** The country of manufacture, formulation, and some mechanical properties of sampled drugs.

Sample code	Formulation	Country of manufacture	Hardness (kPa)	Friability (%)	Disintegration time (s)
AL 1	Tablet	Ghana	3.4 ± 0.30	0.11	671 ± 1.8
AL 2	Tablet	India	3.6 ± 0.10	0.03	611 ± 3.4
AL 3	Tablet	Ghana	5.3 ± 0.02	0.23	554 ± 2.2
AL 4	Tablet	China	4.2 ± 0.01	0.04	671 ± 1.6
AL 5	Tablet	China	4.2 ± 0.13	0.02	791 ± 8.4
AL 6	Tablet	India	3.7 ± 0.21	0.01	866 ± 5.2
AL 7	Suspension	India	—	—	—
AL 8	Suspension	Ghana	—	—	—
AT 1	Injection	India	—	—	—
AT 2	Injection	Ghana	—	—	—

**Table 2:** Uniformity of weight of AL tablets.

Sample code	Weight of 20 tablets/g (*x*)	Average weight/g (*x*/20)	Standard deviation (±)	Deviation by ± 5%	Deviation by > twice ± 5%	Inference
AL 1	5.0840	0.2542	0.0009	0	0	Passed
AL 2	6.9960	0.3498	0.0006	0	0	Passed
AL 3	4.9980	0.2499	0.0029	1	0	Passed
AL 4	5.8020	0.2901	0.0008	0	0	Passed
AL 5	19.4360	0.9718	0.0007	0	0	Passed
AL 6	6.4280	0.3214	0.0003	0	0	Passed

**Table 3:** Weight of samples used in assay.

Sample code	Weight of sample equivalent to 50 mg artemether (g)	Weight of sample equivalent to 100 mg lumefantrine (g)
AL 1	0.6355	0.2118
AL 2	0.8745	0.2915
AL 3	0.6248	0.2083
AL 4	0.7253	0.2418
AL 5	0.6074	0.2025
AL 6	0.8035	0.2678
AL 7	13.8330	4.6108
AL 8	14.190	4.7300

**Table 4:** Percentage content of artemether and lumefantrine in samples.

Sample code	Stated content of artemether (mg)	Actual content of artemether (mg)	Percentage content of artemether (%)	Stated content of lumefantrine (mg)	Actual content of lumefantrine (mg)	Percentage content of lumefantrine (%)	Inference
AL 1	20	20.44	102.18	120	121.38	101.15	Passed
AL 2	20	20.32	101.62	120	120.83	100.69	Passed
AL 3	20	20.24	101.18	120	120.14	100.11	Passed
AL 4	20	19.55	97.74	120	118.45	98.70	Passed
AL 5	80	78.43	98.04	140	140.51	100.36	Passed
AL 6	20	20.38	101.89	120	123.00	102.50	Passed
AL 7	20	20.37	101.84	120	123.02	102.51	Passed
AL 8	20	20.56	102.82	120	134.24	111.87	Failed
AT 1	80	79.94	99.92	—	—	—	Passed
AT 2	80	78.54	98.17	—	—	—	Passed
